# An In Vitro and In Silico Characterization of *Salvia sclarea* L. Methanolic Extracts as Spasmolytic Agents

**DOI:** 10.3390/pharmaceutics15051376

**Published:** 2023-04-29

**Authors:** Milica Randjelović, Suzana Branković, Miloš Jovanović, Nemanja Kitić, Slavoljub Živanović, Tatjana Mihajilov-Krstev, Bojana Miladinović, Milica Milutinović, Dušanka Kitić

**Affiliations:** 1 Department of Pharmacy, Faculty of Medicine, University of Niš, Blvd. Dr Zorana Djindjića 81, 18000 Niš, Serbia; milos.jovanovic@medfak.ni.ac.rs (M.J.); bojana.miladinovic@medfak.ni.ac.rs (B.M.); milica.milutinovic@medfak.ni.ac.rs (M.M.); 2 Department of Physiology, Faculty of Medicine, University of Niš, Blvd. Dr Zorana Djindjića 81, 18000 Niš, Serbia; suzana.brankovic@medfak.ni.ac.rs; 3 Faculty of Medicine, University of Niš, Blvd. Dr Zorana Djindjića 81, 18000 Niš, Serbia; voidruner@gmail.com; 4 Research Center for Biomedicine, Faculty of Medicine, University of Niš, Blvd. Dr Zorana Djindjića 81, 18000 Niš, Serbia; slavoljub.zivanovic@medfak.ni.ac.rs; 5 Department of Biology and Ecology, Faculty of Science and Mathematics, University of Nis, Višegradska 33, 18000 Niš, Serbia; tatjanamk@pmf.ni.ac.rs

**Keywords:** *Salvia sclarea* L., methanolic extracts, spasmolytic activity, ileum, trachea, rats

## Abstract

The use of medicinal plant species and their products is widespread in the field of gastrointestinal and respiratory diseases. This study aimed to evaluate the traditional use of *Salvia sclarea* L., clary sage, finding the possible mechanisms of its spasmolytic and bronchodilator actions in in vitro conditions supported by molecular docking analysis, along with the antimicrobial effects. Four dry extracts were prepared from the aerial parts of *S. sclarea*, using absolute or 80% (*v*/*v*) methanol by the method of a single-stage maceration or an ultrasound-assisted extraction. Characterization of the bioactive compounds by high-performance liquid chromatography indicated the presence of significant amounts of polyphenolics, with rosmarinic acid as the prevalent one. The spontaneous ileal contractions were best inhibited by the extract prepared with 80% methanol and maceration. The same extract was superior in the carbachol- and KCl-induced tracheal smooth muscle contractions, being the strongest bronchodilator agent. The most powerful relaxation of KCl-induced ileal contractions was achieved with the extract made of absolute methanol by maceration, while the 80% methanolic extract made with the ultrasound method generated the best spasmolytic effects in the acetylcholine-induced ileal contractions. Docking analysis suggested that apigenin-7-*O*-glucoside and luteolin-7-*O*-glucoside exhibited the highest binding affinity to voltage-gated calcium channels. Gram (+) bacteria were more susceptible to the effects of the extracts, particularly *Staphylococcus aureus*, in contrast to Gram (−) bacteria and *Candida albicans*. This is the first study to point out the influence of *S. sclarea* methanolic extracts on the gastrointestinal and respiratory spasm reduction, paving the way for their potential place in complementary medicine.

## 1. Introduction

New products harnessing natural sources are rapidly increasing in the market. Following that, studies showing the efficacy and importance of medicinal plant species are being conducted in many countries around the world and cover a wide range of developmental stages [1]. A modern term, coined “reverse pharmacology”, describes a strategy of developing new herbal medicines in modern phytotherapy, by reverse engineering traditional herbal medicine. It involves the study of active compounds based on the traditional use of herbal medicines or formulations; thus, most research on these species relates to the confirmation of their traditional use [2].

The use of medicinal plant species and their products is very widespread in the field of gastrointestinal diseases. The most common indications of herbal drugs and preparations include dyspepsia, constipation, gastro-oesophageal reflux, irritable bowel syndrome, esophagitis, gastritis, and chronic gastrointestinal infections (dysbiosis). They also show properties that are useful in allergies, and with intolerance, peptic ulcers, inflammatory diseases, ulcerative colitis, Crohn’s disease, diverticulitis, as well as anorexia. Aromatic drugs (e.g., from the Lamiaceae and Apiaceae families) are used as excellent spasmolytic and carminative agents and are often used in the cases of colics, flatulence, slow digestion problems, chronic inflammations, and infections [3]. In addition, they are known as natural remedies to respiratory problems due to their broncholytic, secretomotor and spasmolytic effects, and as supportive cough therapy agents [4,5]. A concomitant symptom of many respiratory tract disorders is bronchoconstriction or bronchospasm, which is an abnormal contraction of the smooth muscle airway, thought to result from an intrinsic abnormality in the airway myocytes [6]. Herbal preparations are often used in the treatment or co-treatment of many respiratory diseases, most commonly in inflammation of the upper respiratory organs, and can be helpful in chronic bronchitis and tracheitis, asthma, and chronic obstructive pulmonary disease [3].

Aromatic plants that express significant pharmacological effects from the Lamiaceae family are used in phytomedicine worldwide [7]. Numerous species of the Lamiaceae family belong to the genus of sage, *Salvia* L., whose range of traditional uses in medicine is extremely broad. They are most often used as carminatives and antispasmodics, but also as antiseptic, insecticidal, and anti-inflammatory agents [8]. *Salvia sclarea* L., clary sage, is mostly used in a dry or fresh form as a stomachic for digestive problems thanks to its antispasmodic effect and distinctly aromatic properties. Its usage in traditional medicine is also applied as a mean of relieving respiratory problems, as emmenagogue, hypoglycemic, and a hemostatic agent, in the treatment of menstrual discomfort, gingivitis, polyarthritis, rheumatism, etc. [9,10,11,12]. According to the instructions of Hager’s manual (1994) the aerial parts of *S. sclarea* are used per os for digestive and menstrual problems, general weakness, catarrh, headache, spasms, and kidney problems, and topically for inflammatory wounds. For a per os administration, 4–5 spoons of the drug are used that have to be previously boiled with 0.5 L of water for several minutes. For the treatment of gingivitis, other inflammatory processes in the oral cavity, and for cleaning wounds, the *S. sclarea* tea is prepared in the form of a decoction (50 g of the drug and 1 l of water) by boiling for 1–2 min. Additionally, the manual recommends the combination of *S. sclarea* with vinegar and honey for purulent nail infections [13]. Previous studies confirmed the effectiveness of the essential oil and extracts of *S. sclarea* concerning their antimicrobial, anti-inflammatory, antioxidant, cytotoxic, anticholinesterase, and antidiabetic activities [14,15,16,17,18]. Various secondary metabolites, such as phenolic acids, flavonoids, and terpenes, present in *S. sclarea,* are responsible for these effects [19,20]. Therefore, the aim of this work was to confirm the spasmolytic activities of clary sage methanolic extracts in in vitro conditions relating to the effects of their phytocompounds determined by a docking analysis, along with a study of their antimicrobial effects. The microorganisms selected for testing are the most common causative agents of gastrointestinal and respiratory infections. Spasms in these organ systems are often associated with the infections [21,22], so the spasmolytic activity studies were complemented with the antimicrobial activity test.

## 2. Materials and Methods

### 2.1. Plant Material and Extraction

Aerial parts of *S. sclarea* were collected in the surrounding area of Niš, Malča (Serbia), during the flowering period. The plant material was identified by prof. Dr. Bojan Zlatkovic, from the Department of Biology and Ecology, Faculty of Science, University of Niš. A voucher specimen was deposited in the Herbarium of the Institute of Botany and Botanical Garden “Jevremovac” of the Faculty of Biology, University of Belgrade under collector number 17077.

The plant material was dried in a dark, cool and well-ventilated place and ground to powder on a mechanical mill. The powdered plant material was extracted with absolute and 80% (*v*/*v*) methanol using the ultrasonic technique and the single-stage maceration in the 1:10 ratio. The method of ultrasonic extraction involved the extraction of the drug with a solvent in an ultrasonic bath for 20 min (extracts MU and M80U). The extraction was conducted at room temperature in an ultrasonic bath: Elmasonic S 40 H (220–240 V, 340 W, 37 Hz) (Elma Schmidbauer GmbH, Singen, Germany). The extraction process of the single-stage maceration lasted five days with shaking conducted twice a day, after which the macerate was separated from the rest of the drug by filtering and pressing. The resulting macerate was kept in a cold place for another two days [23] (extracts MM and M80M). The final four extracts were obtained after the filtration and total evaporation of the used solvents in a rotary vacuum evaporator at 40 °C (IKA-Werke GmbH & Co. KG, Staufen, Germany). The extracts were stored in well-closed glass vials at a temperature of up to 4 °C until analysis.

### 2.2. HPLC Characterization of the Extracts

The extracts were chemically characterized on an Agilent 1200 HPLC system (Agilent Technologies, Palo Alto, CA, USA) with a diode array detector. Purospher STAR RP-18e (150 × 4.6 mm) with the particle size of 5 µm (Merck, Darmstadt, Germany) was used as the analytical column. The extracts were primarily dissolved in ultrapure methanol, HPLC grade (10 mg/mL), and filtered through a 0.45 µm microfilter. The extract solutions were injected at a volume of 10 µL. The mobile phase consisted of a mixture of 0.1% aqueous trifluoroacetic acid (A) and acetonitrile (B) with a linear gradient: 0–3 min 5–5% B, 3–32 min 5–28% B, 32–44 min 25–50% B, 44–52 min 50–80% B, 52–54 min 80–90% B, 54–59 min 90–5% B, and 59–60 min 5% B. The flow in the column was 0.7 mL/min and the operating temperature was maintained at 30 °C. Phytocompounds were identified and quantified on the basis of UV-Vis signal response compared to standards. Their quantities in the extracts were expressed as µg/mg [24].

### 2.3. Effects of the Extracts on Ileum and Trachea Contractions

#### 2.3.1. Experimental Animals

All experimental procedures were performed in accordance with the European Directive 2010/63/EU for animal experiments, with the special approval of the Veterinary Directorate of the Ministry of Agriculture and Environmental Protection of the Republic of Serbia (decision number 323-07-00073/2017-05/04). Male Wistar albino rats, which weighed 200–250 g and were 10–12 weeks of age, and were bred in the vivarium of the Faculty of Medicine, University of Niš, were used for all of the experimental series. A week before the experimentations, the animals were separated and housed in stainless steel cages under standard conditions (room temperature 20–24 °C, with a 12 h light/dark regime). The animals had free access to food and water, except for the last 24 h before the experiments, when they were deprived of food.

#### 2.3.2. Isolation and Placement of Ileum and Trachea

After anaesthesia, the thorax and the aorta of the rats were dissected and the ileum and trachea were isolated and cleaned off the mesentery and connective tissue, respectively. The ileal and tracheal fragments were placed in a 20 mL tissue bath, containing Tyrode’s or Krebs’ solution, respectively, and maintained at 37 °C with a constant introduction of a mixture of oxygen (95%) and carbon dioxide (5%). Tyrode’s solution contained NaCl (150 mM), KCl (2.7 mM), MgCl_2_ (2 mM), NaHCO_3_ (12 mM), NaH_2_PO_4_ (0.4 mM), CaCl_2_ (1.8 mM), and glucose (5.5 mM). Kreb’s solution consisted of NaCl (137 mM), KCl (2.81 mM), CaCl_2_ (1.8 mM), MgCl_2_ (0.1 mM), NaH_2_PO_4_ (0.417 mM), NaHCO_3_ (11.9 mM), and glucose (11.10 mM). The fragments were stretched and stabilized for at least 30 min (ileum) or 60 min (trachea) before starting experiments [21]. The changes in the contractility of the organs were recorded using the system Transducer-TSZ-04-E (Experimetria Ltd., Budapest, Hungary). The data were analyzed using the SPEL Advanced ISOSYS Data Acquisition System software.

#### 2.3.3. Experimental Design with Ileum

The first experimental series analyzed the effects of *S. sclarea* methanolic extracts on spontaneous contractions of the isolated rat ileum. The extract solutions were added in cumulative doses in the range from 0.005 to 1.5 mg/mL after the period of adaptation, forming a concentration–response curve. The result of the spasmolytic effect of each extract concentration was expressed as a percentage in relation to the initial spontaneous activity of the isolated ileum (% of inhibition of ileal contractility). Papaverine was used as a positive control with the concentrations of 0.01–3 μg/mL [25].

The second experimental series examined the effects of the extracts on the contractions induced by a high concentration of potassium ions by adding KCl solution (80 mM) after the adaptation period. The tonic contractions of the ileum were followed by the addition of the cumulative doses of each extract solution (0.005–1.5 mg/mL) at 15 min intervals. The relaxations of the ileum smooth muscles, pre-contracted with potassium ions, were expressed as the percentage of the control response mediated by potassium ions. The same procedure was carried out with a calcium channel antagonist, verapamil, with the concentrations range of 0.015–1.5 μg/mL [25].

In the last series, contractions of ileum smooth muscles were stimulated by the cumulative addition of acetylcholine solutions in concentrations of 5, 15, 50, 150, 500, and 1500 nM after a period of adaptation. A control curve of the dose-dependent contractions was constructed according to the obtained results. The ileum segments were washed with Tyrode’s solution until stable spontaneous contractions were established again. Each extract was added at a concentration of 0.5 mg/mL and 1.5 mg/mL to the bath, and after 5 min, a series with the same acetylcholine concentrations was repeated. New curves of the dose-dependent acetylcholine contractions were constructed. The spasmolytic effects of the *S. sclarea* extracts were presented through a series of curves showing the contractile effect of acetylcholine (%) in the presence of the extracts compared with the effect of acetylcholine without them. The same procedure was repeated with atropine (140 nM), a non-selective muscarinic receptor blocker [25].

#### 2.3.4. Experimental Design with Rat Trachea

The first series of experiments studied the effects of the *S. sclarea* methanolic extracts on tracheal contractions caused by carbachol. Carbachol was added at a concentration of 1 μM after spontaneous contractions were established. The extracts were cumulatively added to the organ bath (0.005–1.5 mg/mL). The spasmolytic effects of the extracts were presented as an inhibition percentage of the contractile action of carbachol. Atropine was used as a positive control (0.41–100 μg/mL) [18].

The second set of experiments studied the spasmolytic effects of the extracts on contractions induced by a high concentration of potassium ions (KCl, 80 mM) after the period of stabilization. The extracts were added at the same cumulative concentration. The relaxations of tracheal spasms by the extracts were expressed as a percentage of the contractile action inhibition carried out by the potassium ions. Verapamil was used as a positive control (0.41–100 μg/mL) [18].

### 2.4. Molecular Docking Analysis

Simulation of molecular docking of the ligands to the target protein was performed using the AutoDock Vina software. Dominant phytochemicals, previously determined in *S. sclarea* extracts, and verapamil, as a standard drug, were considered as ligands. The structures of ligands were retrieved from PubChem (https://pubchem.ncbi.nlm.nih.gov, accessed on 20 January 2022) with the following CID numbers: 5281792 rosmarinic acid, 689043 caffeic acid, 5280443 apigenin, 5280445 luteolin, 161271 salvigenin, 12304093 apigenin-7-*O*-glucoside, 45933934 luteolin-7-*O*-glucoside, and 2520 verapamil (standard drug). Guided by the results of in vitro studies that indicate that the spasmolytic activity of *S. sclarea* extract is mainly mediated via calcium-mediated signaling pathways, voltage-gated calcium channels belonging to this signal pathway were selected as the target. The three-dimensional crystal structure of the voltage-gated calcium channel subunit *beta*2a (PDB: 1T0J) used as the target protein was obtained in PDB format from the RCSB Protein Data Bank (https://www.rcsb.org, accessed on 20 January 2022). The target protein and ligands were prepared in a dockable PDBQT format using AutoDock Tools. The coordinates of the grid box center were adjusted in the binding pocket of the standard drug (x: −3.348, y: 3.044, and z: −12.198), while the box dimensions were 20 × 20 × 20 Å. To compare the in silico performance, the binding affinities of the selected ligands with the target molecule were calculated and scored according to their affinity scores calculated through the binding free energy (kcal/mol). The molecular interactions of docked complex ligand–target protein were determined using the AutoDock Vina analysis and visualized with the BIOVIA Discovery Studio Visualizer.

### 2.5. Evaluation of Antimicrobial Activity of the Extracts

The antimicrobial activity of the *S. sclarea* methanolic extracts was estimated with laboratory control strains from the American Type Culture Collection (ATCC). Gram (+) bacteria used in the evaluation were: *Staphylococcus aureus* ATCC 6538, *Enterococcus faecalis* ATCC 9433, *Streptococcus pneumoniae* ATCC 6301, *Streptococcus pyogenes* ATCC19615, *Bacillus cereus* ATCC 11778, and *Lysteria monocytogenes* ATCC 15313. The following bacteria were selected for the testing of the effects of the extracts against Gram (−) representatives: *Pseudomonas aeruginosa* ATCC 9027, *Proteus mirabilis* ATCC 12453, *Salmonela enteritidis* ATCC 13076, *Escherichia coli* ATCC 8739, *Enterobacter aerogenes* ATCC 13048, and *Klebsiella pneumoniae* ATCC 10031. A fungus (yeast), *Candida albicans* ATCC 24433, was used for assessing antifungal activity. The antimicrobial activity of the extracts was evaluated using the microdilution method according to the CLSI (Clinical and Laboratory Standards Institute) [26].

Overnight broth cultures of the tested bacteria and the yeast were used for the preparation of the suspensions, which were adjusted to 0.5 McFarland standard turbidity (corresponded to 1.5 × 10^8^ CFU (colony forming units)/mL for the bacteria and 1.5 × 10^7^ CFU/mL for the yeast). Primarily, the extracts were dissolved in a sterile 10% aqueous solution of dimethyl sulfoxide. Serial double dilutions of the extracts, in the range from 0.1 to 100 mg/mL, were prepared in microtiter plates (96-well) with an inoculated nutrient broth. The final volume of the wells was 100 µL and the final concentrations were 2 × 10^6^ for the bacteria and 2 × 10^5^ for the yeast. The incubation of the microtiter plates was performed at 37 °C for 24 h for the bacteria or at 25 °C for 48 h for the yeast. Microbial growth was detected by a 0.5% aqueous solution of 2,3,5-triphenyl tetrazolium chloride (20 µL) added to each well [27]. The minimal concentration of the extract with no visible growth of microorganisms was defined as the minimum inhibitory concentration (MIC). The minimum bactericidal/fungicidal concentration (MBC/MFC) was defined as the minimal concentration of the extract that killed 99.9% of the tested bacteria or the yeast. For the determination of the MBC/MFC of the extract, the broth was taken from each well with no visible growth and inoculated into an agar (Mueller–Hinton agar at 37 °C for 24 h for the bacteria or Sabouraud dextrose agar 25 °C for 48 h for the yeast). Sterile 10% aqueous DMSO solution was used as a negative control. Chloramphenicol, streptomycin, and nystatin (0.008–16 μg/mL) were used as the controls.

### 2.6. Statistical Analysis

The final results are expressed as mean values of three or six parallel measurements ± the standard deviations (chemical composition or spasmolytic analyses, respectively), except for the antimicrobial activity. The EC_50_ values, which presented the concentrations causing 50% of maximal response, were obtained by a regression analysis. Student’s *t*-test or one-way ANOVA with Duncan’s post hoc test were used for the determination of significant statistical differences between/among the means (*p* < 0.05 or *p* < 0.01). Statistical analyses were carried out using the SPSS 20.0 statistical package (SPSS, Inc., Chicago, IL, USA).

## 3. Results

### 3.1. Chemical Characterization of the Extracts

**Figure 1:**
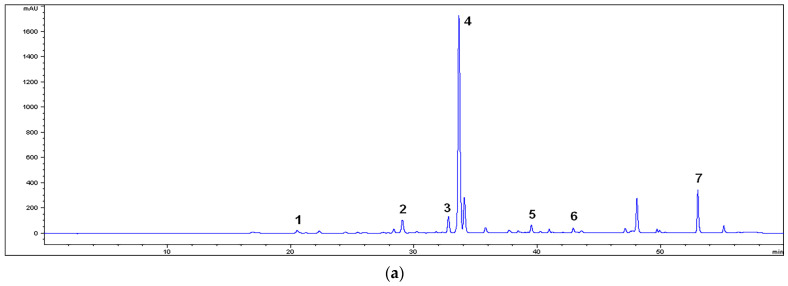

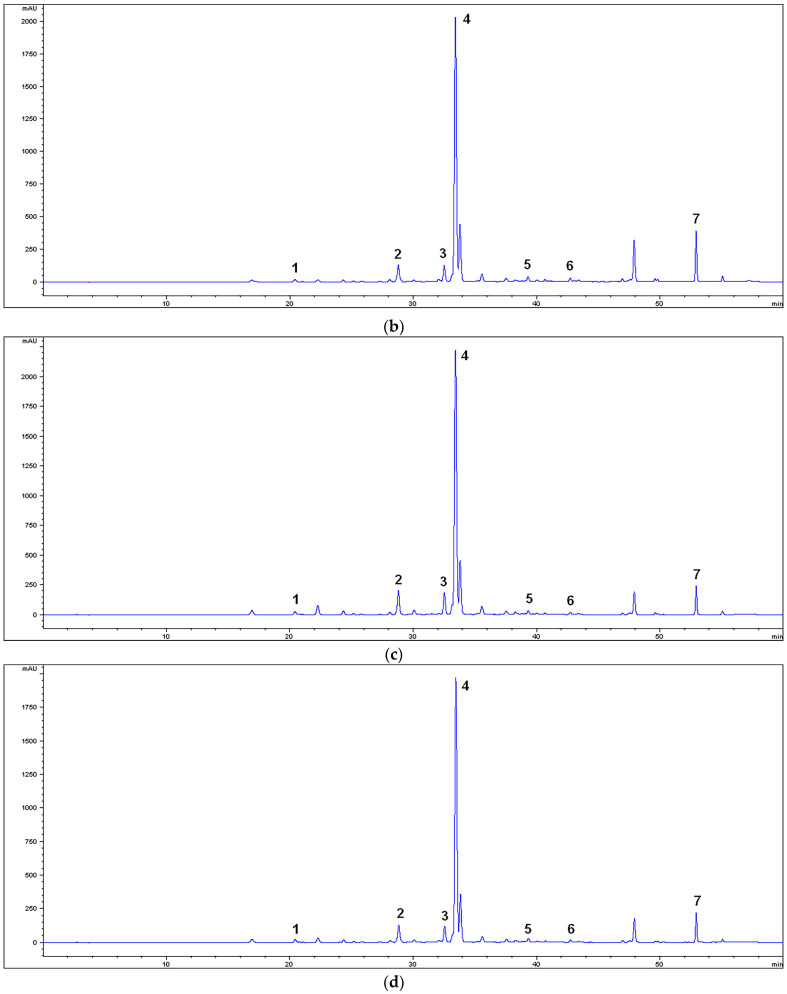
HPLC chromatograms of the *Salvia sclarea* L. extracts ((**a**) MM, (**b**) MU, (**c**) M80M, and (**d**) M80U) (330 nm). 1. Caffeic acid, 2. luteolin-7-*O*-glucoside, 3. apigenin-7-*O*-glucoside, 4. rosmarinic acid, 5. luteolin, 6. apigenin, and 7. salvigenin.

The yields of the extractions were 12.75% for the MM extract, 11.93% for MU, 19.65% for M80M, and 13.30% for M80U. The HPLC analysis of the extracts indicated the presence of phenolic acids as well as flavon type flavonoid aglycons and flavon type flavonoid heterosides (Figure 1). Table 1 displays the content of the individual compounds. Rosmarinic acid was predominant in all of the extracts in the range of 171.99 ± 1.88–197.48 ± 2.00 µg/mg. The M80M extract contained the highest quantity of phenolic acids and flavonoid heterosides, while aglycons were mostly present in the extracts prepared with absolute methanol. Dominant aglycon in the extracts was salvigenin, followed by luteolin and apigenin.

Based on the obtained values of the content of phytocompounds in the extracts, the maceration method generally proved to be more effective. This difference is especially noticeable between the extracts prepared with 80% methanol.

### 3.2. Spasmolytic Effects of the Extracts on Spontaneous Ileum Contractions

**Figure 2:**
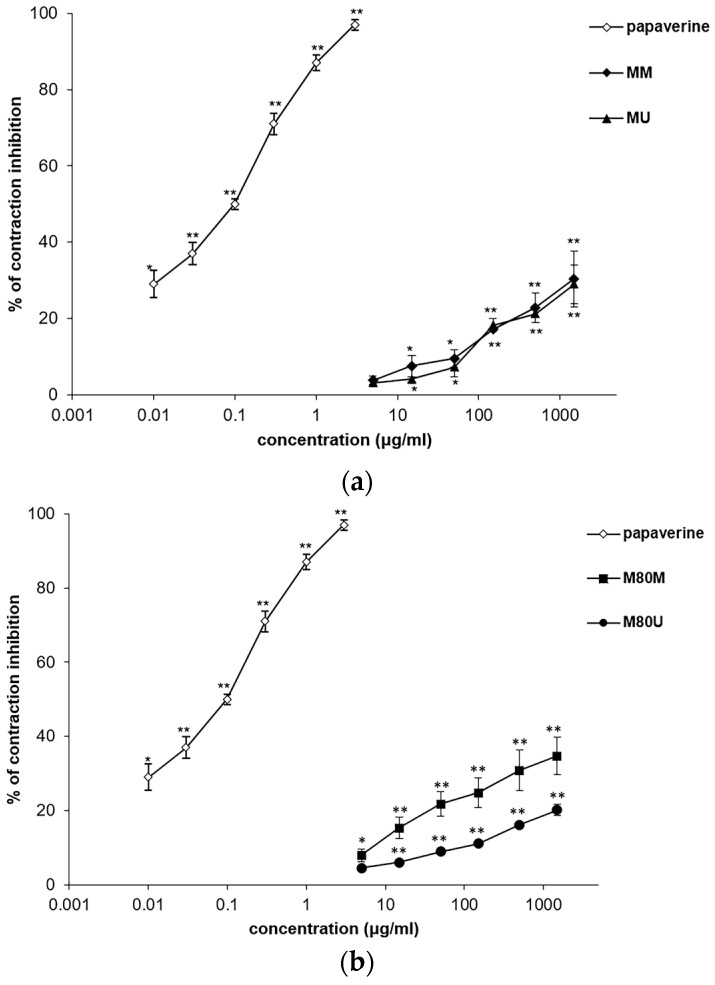
Inhibitory effects of the *Salvia sclarea* L. extracts and papaverine on spontaneous contractions of the isolated rat’s ileum: (**a**) effects of the MM and MU extracts and papaverine; (**b**) effects of the M80M and M80U extracts and papaverine. Each point represents the mean value of percentages with respect to the spontaneous contractions in the Tyrode solution (control) ± SD of six segments (Student’s *t*-test, * *p* < 0.05, ** *p* < 0.01 vs. Tyrode).

The *S. sclarea* methanolic extracts exhibited the significant, dose-dependent, and spasmolytic effect of the ileum smooth muscle in the first experimental series by reducing the spontaneous contractions. The lowest EC_50_ value was determined for the extract M80M. In addition, this extract, MM, and MU acted in a similar manner with the narrow range of EC_50_ from 2.44 ± 0.10 to 2.69 ± 0.22 mg/mL and the inhibitions of maximal concentrations (1.5 mg/mL) from 28.96 ± 1.86 to 34.73 ± 1.20%. The M80U extract was less effective (Table 2; Figure 2). The maximal concentration of papaverine (0.003 mg/mL), used as a positive control, was able to reduce 97% of all spontaneous ileum contractions.

### 3.3. Spasmolytic Effects of the Extracts on KCl-Induced Ileum Contractions

**Figure 3:**
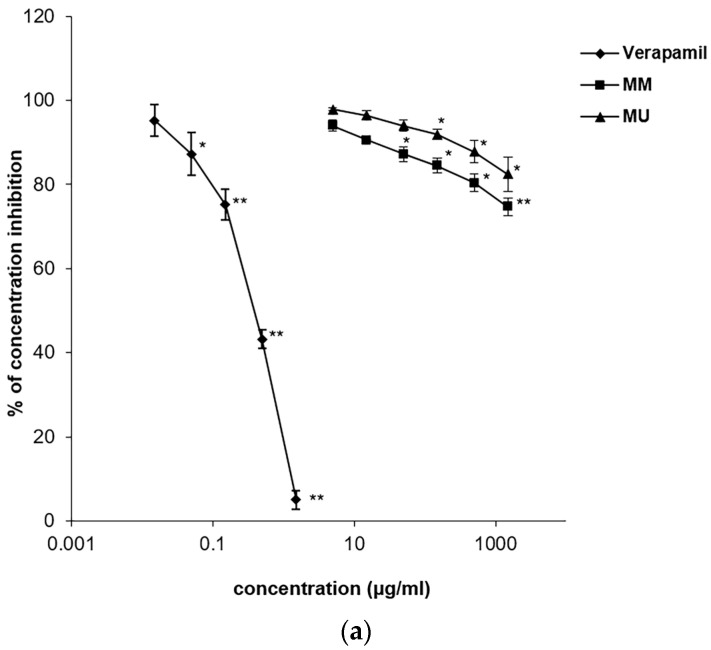

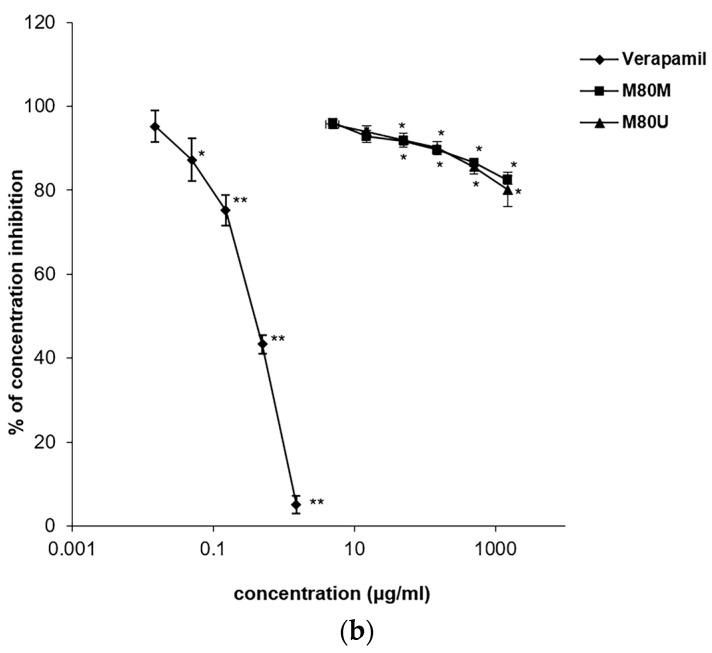
Inhibitory effects of the *Salvia sclarea* L. extracts and verapamil on the KCl-induced contractions of the isolated rat’s ileum: (**a**) effects of the MM and MU extracts and verapamil; (**b**) effects of the M80M and M80U extracts and verapamil. Each point represents the mean value of percentages of maximal response ± SD of six segments (Student’s *t*-test, * *p* < 0.05, ** *p* < 0.01 vs. control).

The extracts had an inhibitory activity on the contractions induced by the application of KCl solution (80 mM). Ileum smooth muscle relaxation was dose-dependent, with the range of EC_50_ values from 3.69 ± 0.30 to 5.76 ± 0.34 mg/mL (Table 2; Figure 3). The best activity was achieved after the addition of the MM extract, whose maximal concentration of 1.5 mg/mL was able to reduce contraction to 74.71 ± 2.29%. Verapamil was used as a control in this study, whereby a maximum concentration of 0.0015 mg/mL reduced the contraction to 5%.

### 3.4. Spasmolytic Effects of the Extracts on Acetylcholine-Induced Ileum Contractions

**Figure 4:**
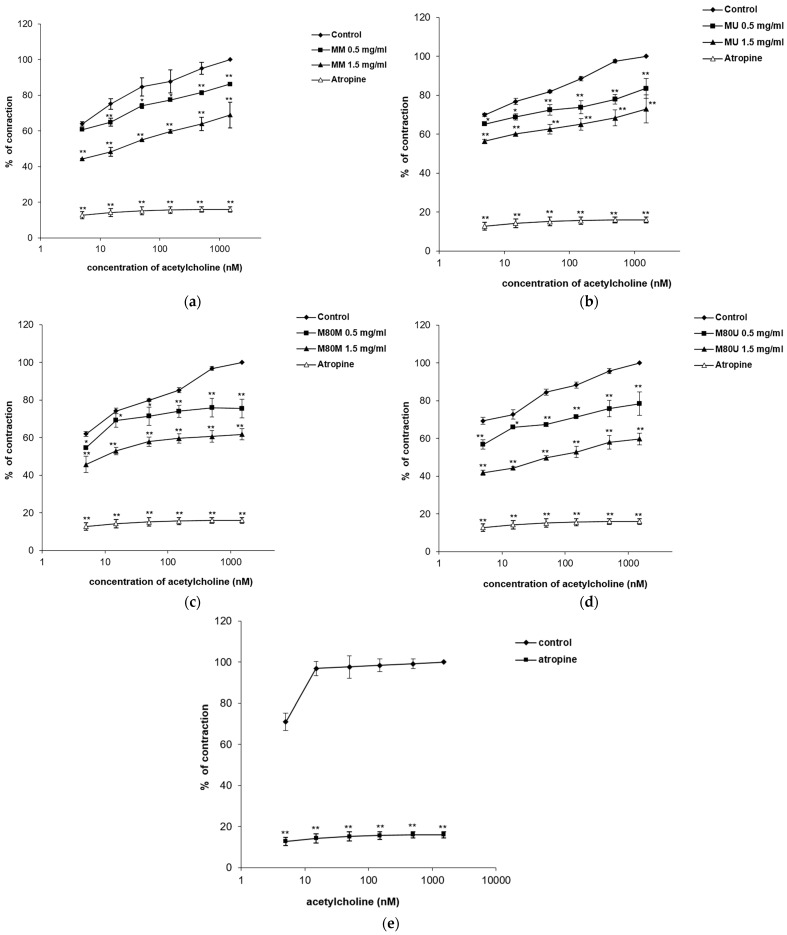
Inhibitory effects of the *Salvia sclarea* L. extracts and atropine on the acetylcholine (Ach)-induced contractions of the isolated rat’s ileum: (**a**) the values of control, Ach + MM (0.5 mg/mL) and Ach + MM (1.5 mg/mL); (**b**) the values of control, Ach + MU (0.5 mg/mL), and Ach + MU (1.5 mg/mL); (**c**) the values of control, Ach + M80M (0.5 mg/mL), and Ach + M80M (1.5 mg/mL); (**d**) the values of control, Ach + M80U (0.5 mg/mL), and Ach + M80U (1.5 mg/mL); and (**e**) the values of control, Ach + atropine (140 nM). Each point represents the mean value of percentages of maximal response ± SD of six segments (Student’s *t*-test, * *p* < 0.05, and ** *p* < 0.01 vs. control).

The tested extracts were able to reduce ileum contractions induced by cumulative doses of acetylcholine with statistical significance (*p* < 0.01). The control EC_50_ values of acetylcholine were modified and increased after the addition of all the extract concentrations (0.5 and 1.5 mg/mL) (Table 3; Figure 4). The M80U extract stood out in this series of experiments modifying the baseline EC_50_ value of acetylcholine (0.17 ± 0.00 nM) two times after the application of 0.5 mg/mL. An even greater increase in the EC_50_ value of acetylcholine was observed after applying a dose of 1.5 mg/mL (62.15 ± 3.22 nM and contractions reduction from baseline 100% to 59.78 ± 3.10%). The effects of the other methanolic extracts were significantly lower: M80M > MM > MU. Atropine, a muscarinic receptor antagonist, was used as a positive control in the concentration of 140 nM, modifying the EC_50_ value of acetylcholine from 0.10 ± 0.00 nM to 18,261.96 ± 958.32 nM and reducing acetylcholine-induced ileum contractions to 16.02%.

### 3.5. Spasmolytic Effects of the Extracts on KCl-Induced Tracheal Contractions

**Figure 5:**
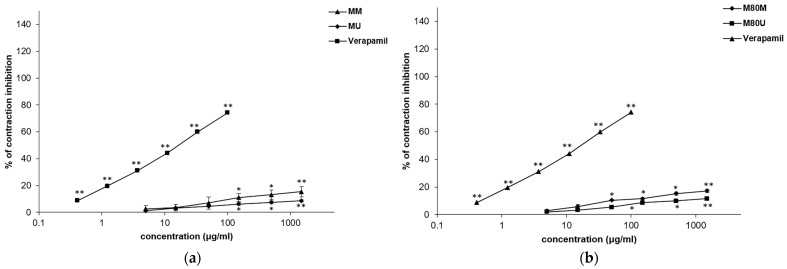
Inhibitory effects of the *Salvia sclarea* L. extracts and verapamil on the KCl-induced contractions of the isolated rat trachea: (**a**) effects of the MM and MU extracts and verapamil; (**b**) effects of the M80M and M80U extracts and verapamil. Each point represents the mean value of percentages of inhibitions ± SD of 6 segments (Student’s *t*-test, * *p* < 0.05, and ** *p* < 0.01 vs. control).

After a single dose of KCl (80 nM) all tested extracts expressed inhibitory effects on tracheal rat smooth muscle contractions in a dose-dependent regime (Table 4). Samples were characterized as moderate spasmolytic agents, whereby extracts prepared by the maceration method were stronger, especially M80M. A maximal concentration of 1.5 mg/mL of this extract inhibited the contractions by 15.37 ± 0.81 and 17.22 ± 0.99%, respectively (Figure 5). Verapamil inhibited 74.23 ± 1.20% of the contractions at the maximum concentration (100 μg/mL) with an EC_50_ value of 15.23 ± 0.08 μg/mL.

### 3.6. Spasmolytic Effects of the Extracts on Carbachol-Induced Tracheal Contractions

**Figure 6:**
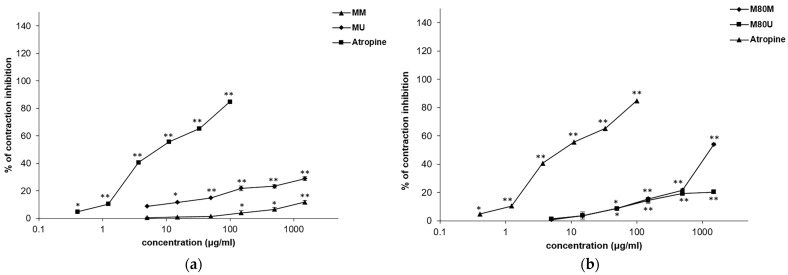
Inhibitory effects of the Salvia sclarea L. extracts and atropine on the carbachol-induced contractions of the isolated rat trachea: (**a**) effects of the MM and MU extracts and atropine; (**b**) effects of the M80M and M80U extracts and atropine. Each point represents the mean value of percentages of inhibitions ± SD of six segments (Student’s *t*-test, * *p* < 0.05, and ** *p* < 0.01 vs. control).

Methanolic S. sclarea extracts had a relaxing effect on the tracheal smooth muscle contractions induced by a single dose of carbachol (1 μM) (Table 4; Figure 6). The M80M extract stood out in in its series with the lowest EC_50_ (1.36 ± 0.01 mg/mL) and an inhibition of 54.09 ± 1.66% achieved with a maximum concentration of 1.5 mg/mL. Atropine, used as a control, inhibited 84.89 ± 2.00% of the tracheal contractions with an EC_50_ value of 9.78 ± 0.00 μg/mL.

### 3.7. Molecular Docking Analysis

**Figure 7:**
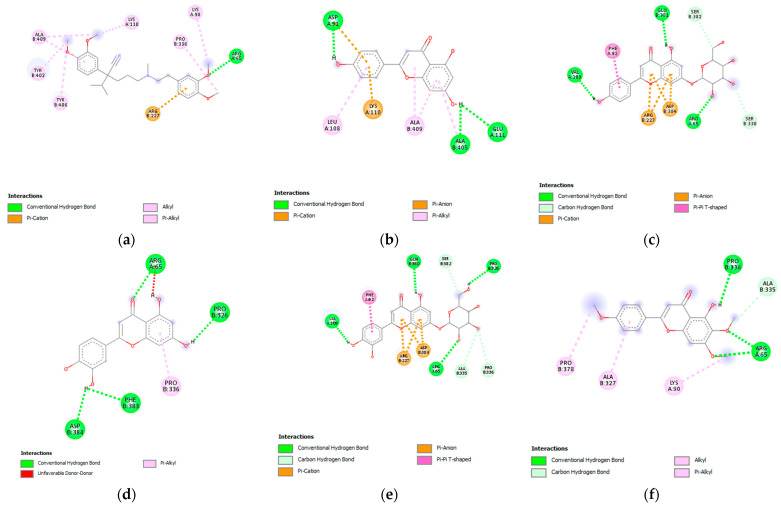

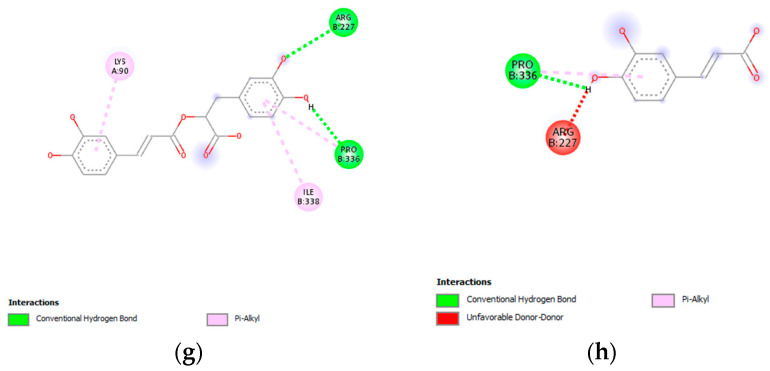
Visualization of the molecular interactions of voltage-gated calcium channels with phytocompounds of the *Salvia sclarea* L. extract: (**a**) verapamil; (**b**) apigenin; (**c**) apigenin-7-*O*-glucoside; (**d**) luteolin; (**e**) luteolin-7-*O*-glucoside; (**f**) salvigenin; (**g**) rosmarinic acid; and (**h**) caffeic acid.

The results of the molecular docking analysis with the binding affinities and residues of the amino acids involved in the ligand–target binding, including their interatomic distance (Å), are listed in Table 5. The results show that the docking scores of all tested ligands are negative, with the binding energies in the range from −5.8 to −8.6 kcal/mol. This suggests that ligands are able to bind to the target protein. The docking score of the tested ligands with voltage-gated calcium channels is as follows: apigenin-7-*O*-glucoside = luteolin-7-*O*-glucoside, salvigenin, luteolin, apigenin, rosmarinic acid = verapamil, and caffeic acid. The most prominent apigenin-7-*O*-glucoside and luteolin-7-*O*-glucoside both had a binding score of −8.6 kcal/mol. For a deeper analysis of the interaction profile, two-dimensional structures of the binding pocket of the target protein in a complex with tested ligands were constructed (Figure 7). The analysis of the interaction profile shows that in the case of both mentioned compounds binding via conventional hydrogen bonds to the amino acid residue, Arg65 is involved, similar to the binding of the standard drug verapamil to its binding pocket. In the case of apigenin-7-*O*-glucoside, the length of this bond was 2.21 Å; while in the case of luteolin-7-*O*-glucoside, it was slightly longer. Apart from this hydrogen bond interaction, apigenin-7-*O*-glucoside forms conventional hydrogen bonds with the residues of Val109 and Glu381, while luteolin-7-*O*-glucoside forms conventional hydrogen bonds with Pro326, Val109, and Gln380. Such a developed hydrogen bond network contributes greatly to the binding affinity value. Regarding the hydrophobic/electrostatic interactions, both apigenin- and luteolin-7-*O*-glucoside achieved a π-cation interaction via Arg227 such as verapamil. Beyond, both apigenin- and luteolin-7-*O*-glucoside showed a poorer network of hydrophobic interactions compared to verapamil (Table 5).

### 3.8. Antimicrobial Activity of the Extracts

According to the obtained results of the MIC and MBC/MFC values, the extracts are moderate antimicrobial agents (Table 6). The effects were more prominent toward Gram (+) bacteria compared to Gram (−). In addition, their bactericidal effects were almost minor with the MBC values of 100 or >100 mg/mL, with the exception of the M80M extract, which had somewhat stronger effects on *P. aeruginosa* (50 mg/mL). The MU extract, prepared with absolute methanol and the ultrasound method, showed the best antimicrobial effectiveness, particularly toward *S. aureus*, *B. cereus*, and *L. monocytogenes*, with the MIC values of 6.25, 12.5, and 25 mg/mL, respectively. Its bacteriostatic effects against Gram (−) bacteria were mostly expressed toward *P. aeruginosa* and *E. aerogenes* with the MIC values of 50 mg/mL. The effects on the yeast, *C. albicans*, were of no great importance because the MIC and MFC values of the extracts were 100 and/or >100 mg/mL. The DMSO aqueous solution showed no activity against the investigated microbial strains. MICs/MBCs values of the used positive controls were expectedly much lower (chloramphenicol 0.06–7.81/0.12–15.61 µg/mL, streptomycin 0.16–0.6/0.16–0.6 µg/mL, and nystatine 3.91–7.81 µg/mL).

## 4. Discussion

Many factors of plant extraction have important roles in its efficiency, primarily the type of solvent and method of extraction. The most commonly used methods include conventional techniques, such as maceration, percolation, infusion, decoction, or hot continuous extraction. However, in the last three decades, various new, alternative techniques, such as ultrasonic and microwave extraction, or supercritical fluid extraction, were developed [28]. We used the ultrasound-assisted extraction because it is a technique that generally achieves high reproducibility in a short period of time and a high yield of bioactive compounds. Additionally, this technique is characterized by its simplicity, a low temperature during processing, and a reduced consumption of solvents and energy [29]. On the other hand, the disadvantage of this extraction technique is the risk of possible free radicals formation when the ultrasound energy exceeds 20 kHz [29,30]. The ultrasound method was shown to be very good in terms of the extraction yield in previous studies, although some studies show it to be weaker than Soxhlet extraction, maceration, or microwave extraction [31,32]. Another extraction technique used is single maceration. It is a traditional method that involves the extraction of plant material at room temperature for a minimum of three days [30]. Its advantage is reflected in the use of a cold solvent, which reduces the possibility of the decomposition of active compounds. The content of phytocompounds in the extracts indicates that the maceration method was more effective. The extract prepared with this method, using a more polar solvent (80% methanol), contained the highest quantity of phenolic acids and flavonoid heterosides. In addition, absolute methanol extracted higher amounts of flavonoid aglycones, compared to 80% methanol, due to its lower polarity.

Functional gastrointestinal disorders include a number of morphological and physiological disorders characterized by impaired intestinal motility, visceral hypersensitivity, changes in mucosal function, immune system, altered microbiota, and nervous system processes [33]. The term “functional” generally refers to disturbances in the neuromuscular function of the affected part of the gastrointestinal tract that induce discomfort [33,34]. The *Salvia* species was traditionally used in the therapy of various gastrointestinal complaints [35]. In addition, the European Medicinal Agency published a monograph on *S. officinalis* leaf, indicating its use as traditional herbal medicine in mild dyspeptic complaints, such as heartburn and bloating [36]. Previous studies confirmed that other *Salvia* species could be potential therapeutic agents in functional gastrointestinal disorders as well [37,38,39,40]. In this study, methanolic *S. sclarea* extracts exhibited inhibitory effects to a lesser or greater extent in all experimental models performed on the isolated rat ileum. The contractions of smooth ileum muscles are the result of the increased concentration of free calcium ions in the cytoplasm, which is achieved through voltage-dependent L-type channels or by the release from the intracellular depot [41]. Spontaneous contractions of the smooth ileal muscle were effectively reduced with EC_50_ ranging from 2.44 ± 0.10 mg/mL to 4.59 ± 0.33 mg/mL. The weakest activity was achieved with the M80U extract, which was characterized by the lowest concentration of rosmarinic acid, flavonoids aglycons, and flavonoids heterosides. The other three extracts exhibited similarly in spasmolytic activity. Furthermore, the extracts were able to reduce the contractions stimulated by a high single dose of the KCl solution (80 mM). Smooth muscle contractility is triggered by the depolarization of the smooth muscle membrane, which is caused by high concentrations of potassium ions opening the voltage-dependent L-type channels, causing extracellular calcium ions to enter the cell and cause the contraction [42]. Therefore, the inhibitory effects of the tested extracts on the potassium ion-induced contractions can be explained by the blocking of voltage-dependent L-type calcium channels and the opening of potassium channels. The extract prepared with absolute methanol and maceration turned out to be the best, having the highest amount of apigenin and luteolin. The third series of experiments conducted on the rat ileum showed the inhibitory effects of *S. sclarea* methanolic extracts on contractions induced by acetylcholine, suggesting that the effects of the extracts are mediated by the action on the muscarinic receptors. It is known that the gastrointestinal tract has an abundance of M_2_ and M_3_ muscarinic receptor subtypes, whereby the M_2_ subtype is more prevalent. The acetylcholine neurotransmitter causes the contractions of the isolated ileum primarily through the M_3_ receptor pathway, which involves the hydrolysis of phosphoinositol and the mobilization of intracellular calcium ions [43], as well as the opening of voltage-dependent calcium L-type channels [44]. The tested extracts, in both concentrations (0.5 and 1.5 mg/mL), were able to reduce the smooth muscle contractions induced by cumulative doses of acetylcholine. While it was the weakest in reducing spontaneous and KCl-induced contractions, the M80U extract was the strongest in this series of experiments. This extract modified the baseline EC_50_ value of acetylcholine two times after the application of the first dose and over three hundred times after applying the dose of 1.5 mg/mL. The effects of the other methanolic extracts were lower and were distributed in the following manner: M80M > MM > MU.

The phytocompounds, determined in the *S. sclarea* extracts, are likely to be highly responsible for the aforementioned effects. Results of the in vitro studies suggest that the spasmolytic activity of *S. sclarea* methanolic extract is mediated via calcium-mediated signaling pathways. The seven major phytocompounds of this extract were docked to a voltage-gated calcium channel belonging to this signal pathway [45], using verapamil as the standard drug. A higher negative value of binding energies presented in Table 5 indicates that the ligand could bind to the protein stronger [46]. Concerning binding affinities, it is observed that the most prominent were apigenin-7-*O*-glucoside and luteolin-7-*O*-glucoside, both with a binding score of −8.6 kcal/mol. The length of the conventional hydrogen bond with amino acid residue Arg65 of 2.21 Å in the case of apigenin-7-*O*-glucoside can be classified as strong (in the range of 2.2 to 2.5 Å), while the distance of 2.52 Å in the case of luteolin-7-*O*-glucoside can be categorized as medium strong (within the range of 2.5 to 3.2 Å). The more developed network of hydrophobic interactions observed for verapamil compared to apigenin- and luteolin-7-*O*-glucoside could partially explain its better activity recorded in in vitro assays. Namely, although hydrophobic interactions contribute less to binding energy compared to hydrogen bonds, because they are not as strong, they often play an important role in many biological mechanisms [47].

In addition to the methanol extracts of *S. sclarea*, the inhibiting effects of excellent smooth muscle ileum contractions were reported for the *S. sclarea* ethanolic extracts and essential oil [18,48]. Rosmarinic acid, as the prevalent compound in the extracts, was proven to be an effective spasmolytic agent in in vitro conditions. Bazylko et al. (2009) [49] and Randjelovic et al. (2022) [18] confirmed its activity in spontaneous KCl- and acetylcholine-induced ileum contractions. In addition, Lemmens-Gruber et al. (2006) [50] and Abdalla et al. (1994) [51] showed that apigenin and luteolin exhibit excellent spasmolytic activities on isolated guinea pig ileum. Furthermore, these flavones significantly inhibit the smooth muscle contractility of rat ileum induced by high potassium concentrations, while apigenin also had an effect on reducing acetylcholine-induced contractions [52].

Many studies showed that the extracts and essential oils of plant species of the Lamiaceae family exhibit inhibitory effects on the isolated trachea of experimental animals, and it is well known that these aromatic drugs are traditionally widely used in respiratory disorders of this type [53,54]. The tested *S. sclarea* extracts had a moderate relaxing effect on tracheal smooth muscle contractions caused by a single dose of KCl and carbachol. The best spasmolytic effects in both systems were achieved by the M80M extract, which is characterized by the highest quantity of the more polar compounds: rosmarinic acid, caffeic acid, and luteolin- and apigenin-7-*O*-glucoside. As shown in previous studies, the compounds determined in these extracts exhibited bronchodilator activity. Namely, rosmarinic acid, luteolin, and apigenin were able to inhibit the contractions of tracheal smooth muscle induced by various spasmogenic agents [51,55,56,57].

Aromatic plant species were long recognized and widely used as antibacterial, antifungal, antiviral, or antiparasitic agents [58]. Plant polyphenols are characterized by good antimicrobial properties and their presence in extracts significantly contributes to the inhibition of growth and destruction of microorganisms [59]. According to numerous in vitro studies, polyphenols, identified in the tested extracts, exhibit such effects [18,60,61,62,63,64]. *Salvia* species, along with *S. sclarea* methanolic extracts, are stronger antimicrobial agents toward Gram (+) compared to Gram (−) bacteria, which also applies to most plants [65]. The explanation for this fact lies in the structure of the cell wall of Gram (−) bacteria, which is more complicated and represents a specific barrier for the entry of macromolecules [66]. Furthermore, our investigation confirmed that the *S. sclarea* methanolic extracts had better effects on inhibiting the growth of tested bacteria than killing them, which was also shown by the *S. sclarea* ethanolic extracts [18]. It is of particular importance that the investigated extracts were active against *S. aureus,* keeping in mind its great pathogenicity, especially in regards to its effect on respiratory organs [67]. The MU extract, characterized with the highest quantity of apigenin and salvigenin, was leading in its activity with the MIC value of 6.25 mg/mL. In addition, the extracts showed a significant antimicrobial effect against *B. cereus*, responsible for foodborne illnesses, followed by nausea, vomiting, and diarrhea [68]. The MU and M80U extracts stood out in their activity, with both having MIC values of 12.5 mg/mL. The significance of the bacteriostatic activity of the extracts, especially MM and MU, against *L. monocytogenes*, is that this bacterium is a cause of serious infection, listeriosis, which is characterized by an occasional febrile gastroenteritis in immunocompetent persons and even by a possible fatal outcome [69]. Recent studies showed that the *S. sclarea* extracts could be useful as antimicrobial agents against other Gram (+) bacteria, such as *S. epidermidis*, *B. megaterium*, *B. brevis*, *Micrococcus luteus,* and *Mycobacterium smegmatis* [70,71]. Among Gram (−) bacteria, *P. aeruginosa* and *E. aerogenes* showed a mildly higher sensitivity to the presence of the extracts. Namely, the MU and M80M extracts acted as anti-pseudomonal agents with MIC values of 50 mg/mL, which is important because *P. aeruginosa* is the cause of opportunistic and hospital-acquired infections, having a high resistance to antibiotics [72]. Previous investigations of the antimicrobial characteristics of *S. sclarea* extracts toward Gram (−) bacteria demonstrated their inhibitory effects on *P. mirabilis*, *S. enteritidis*, *K. pneumoniae*, *E. coli*, and *Aeromonas hydrophila* growths [18,71,73]. The investigated extracts did not prove to be effective against the yeast *C. albicans* with values of MICs/MFCs over 100 mg/mL, which was in accordance with other studies of antifungal properties of *S. sclarea* extracts [73,74].

## 5. Conclusions

The present study offers a deeper understanding of the gastrointestinal and bronchodilator activity and the potential use of the *S. sclarea* methanolic extracts in phytotherapy. The extracts successfully reduced spontaneous and induced ileum and tracheal contractions in in vitro conditions. Polyphenolic compounds determined in the extracts, rosmarinic and caffeic acid, apigenin, luteolin, salvigenin, and luteolin- and apigenin-7-*O*-glucoside could be responsible for the manifestation of the spasmolytic activity, which was supported by the in silico analysis. The antibacterial effects of the extracts were moderate, being better bacteriostatic than bactericidal agents; however, they could be supportive agents in the control of gastrointestinal and respiratory disorders.

The investigated *S. sclarea* extracts might be used as potential herbal remedies, although further studies should be aimed at their efficacy in clinical trials.

## Figures and Tables

**Table 1:** Chemical composition of the *Salvia sclarea* L. extracts determined by HPLC.

Compounds	RT (min)	MM	MU	M80M	M80U
		µg/mg			
caffeic acid	20.5	0.79 ± 0.02 ^a^	0.63 ± 0.04 ^b^	0.97 ± 0.07 ^b^	0.81 ± 0.01 ^a^
luteolin-7-*O*-glucoside	29.0	5.18 ± 0.21 ^a^	5.65 ± 0.34 ^a^	8.84 ± 0.54 ^b^	5.61 ± 0.31 ^a^
apigenin-7-*O*-glucoside	32.5	5.63 ± 0.21 ^a^	4.56 ± 0.02 ^b^	7.00 ± 0.55 ^c^	4.45 ± 0.18 ^d^
rosmarinic acid	33.5	175.66 ± 2.02 ^a^	177.77 ± 1.89 ^a^	197.48 ± 2.00 ^b^	171.99 ± 1.88 ^c^
luteolin	39.3	1.45 ± 0.01 ^a^	1.13 ± 0.01 ^b^	0.96 ± 0.02 ^c^	0.80 ± 0.02 ^d^
apigenin	42.7	0.78 ± 0.01 ^a^	0.78 ± 0.00 ^a^	0.72 ± 0.00 ^b^	0.53 ± 0.00 ^c^
salvigenin	53.0	3.72 ± 0.09 ^a^	4.05 ± 0.02 ^b^	2.54 ± 0.04 ^c^	2.27 ± 0.01 ^d^

RT: retention time. The results represent the mean of the three measurements ± the standard deviation. Different lowercase letters in the rows indicate a statistically significant difference in compound content among extracts (Duncan’s test, *p* < 0.05).

**Table 2:** Spasmolytic effects of the *Salvia sclarea* L. extracts and controls on the spontaneous and KCl-induced ileum contractions.

	EC_50_	
	Spontaneous Contractions	KCl-Induced Contractions
	mg/mL	
MM	2.62 ± 0.24 ^a^	3.69 ± 0.30 ^a^
MU	2.69 ± 0.22 ^a^	4.90 ± 0.33 ^b^
M80M	2.44 ± 0.10 ^a^	5.76 ± 0.34 ^c^
M80U	4.59 ± 0.33 ^b^	4.63 ± 0.26 ^b^
papaverine	1.2 × 10^−4^ ± 0.1 × 10^−4 c^	/
verapamil	/	6.3 × 10^−4^ ± 0.5 × 10^−4 d^

The results represent the mean of the three measurements ± the standard deviation. Different lowercase letters in columns indicate a statistically significant difference in EC_50_ values among extracts and controls (Duncan’s test, *p* < 0.05).

**Table 3:** The EC_50_ values of acetylcholine without extracts and atropine (control), the EC_50_ values of acetylcholine with the addition of the *Salvia sclarea* L. extracts in concentrations of 0.5 mg/mL and 1.5 mg/mL, and the EC_50_ values of acetylcholine with the addition of atropine (140 nM).

	MM	MU	M80M	M80U	Atropine
EC_50_ of Acetylcholine (nM)					
control	0.29 ± 0.01 ^a^	0.03 ± 0.00 ^a^	0.01 ± 0.00 ^a^	0.17 ± 0.00 ^a^	0.10 ± 0.00 ^a^
0.5 mg/mL	0.40 ± 0.01 ^b^	0.12 ± 0.00 ^b^	0.15 ± 0.00 ^b^	0.36 ± 0.00 ^b^	/
1.5 mg/mL	18.21 ± 0.65 ^c^	0.47 ± 0.01 ^c^	7.25 ± 0.22 ^c^	62.15 ± 3.22 ^c^	/
140 nM	/	/	/	/	18,261.96 ± 958.32 ^b^

The results represent the mean of six measurements ± standard deviation. Different lowercase letters in columns indicate statistically significant difference in EC_50_ values among control and different extracts (positive control) concentrations (Duncan’s test, *p* < 0.05).

**Table 4:** Spasmolytic effects of the *Salvia sclarea* L. methanolic extracts and controls on the KCl-and carbachol-induced tracheal contractions.

	EC_50_	
	KCl-Induced Contractions	Carbachol-Induced Contractions
	mg/mL	
MM	6.27 ± 0.16 ^a^	6.92 ± 0.04 ^a^
MU	15.38 ± 1.02 ^b^	3.26 ± 0.02 ^b^
M80M	6.03 ± 0.33 ^a^	1.36 ± 0.01 ^c^
M80U	9.02 ± 0.11 ^c^	4.26 ± 0.04 ^d^
verapamil	1.53 × 10^−2^ ± 8.00 × 10^−5 d^	/
atropine	/	9.78 × 10^−3^ ± 0.00 ^f^

The results represent the mean of the three measurements ± the standard deviation. Different lowercase letters in columns indicate a statistically significant difference in compound content among extracts and controls (Duncan’s test, *p* < 0.05).

**Table 5:** Docking score of dominant compounds in the *Salvia sclarea* L. methanolic extract considered as ligands and voltage-gated calcium channel.

Compounds	Binding Affinity (kcal/mol)	Hydrogen Bonds	Electrostatic/Hydrophobic Bonds
V	−6.6	Conventional hydrogen bond: Arg65 (2.36)	π-Cation: Arg227 (4.23) Alkyl: Lys110 (4.12), Ala409 (4.50), Lys90 (4.13), Pro336 (4.38) π-Alkyl: Ala409 (5.06), Tyr402 (4.64), Tyr406 (5.00)
A	−6.7	Conventional hydrogen bond: Asp91 (2.05), Glu111 (2.86), Ala405 (2.30)	π-Cation, π-donor hydrogen bond: Lys110 (2.92) π-Anion: Asp91 (4.07) π-Alkyl: Ala409 (5.37), Ala405 (5.31), Ala409 (4.06), Leu108 (5.34), Lys110 (4.22)
AG	−8.6	Conventional hydrogen bond: Val109 (2.09), Glu381 (2.33), Arg65 (2.21) Carbon hydrogen bond: Ser382 (3.65), Ser330 (3.49)	π-Cation: Arg227 (4.24), Arg227 (4.26) π-Anion: Asp384 (3.85), Asp384 (3.84) π-π T-shaped: Phe92 (4.83)
L	−6.8	Conventional hydrogen bond: Phe383 (2.49), Asp384 (2.55), Pro326 (2.47), Arg65 (2.65)	π-Alkyl: Pro336 (4.98)
LG	−8.6	Conventional hydrogen bond: Pro326 (2.61), Val109 (1.82), Gln380 (2.45), Arg65 (2.52) Carbon hydrogen bond: Ser382 (3.56), Ala335 (3.64), Pro336 (3.67)	π-Cation: Arg227 (4.17), Arg227 (4.00) π-Anion: Asp384 (3.40), Asp384 (3.96) π-π T-shaped: Phe92 (4.82)
S	−6.9	Conventional hydrogen Bbond: Pro336 (2.49), Arg65 (2.70), Arg65 (2.14) Carbon Hydrogen Bond: Ala335 (3.51)	Alkyl: Pro378 (4.61), Lys90 (3.85) π-Alkyl: Ala327 (5.26)
RA	−6.6	Conventional hydrogen bond: Pro336 (2.72), Arg227 (2.74), Arg227 (2.95)	π-Alkyl: Pro336 (5.05), Ile338 (5.35), Lys90 (4.60)
CA	−5.8	Conventional hydrogen bond: Pro336 (2.11)	π-Alkyl: Pro336 (4.89)

V—verapamil; A—apigenin; AG—apigenin-7-*O*-glucoside; L—luteolin; LG—luteolin-7-*O*-glucoside; S—salvigenin; RA—rosmarinic acid; and CA—caffeic acid.

**Table 6:** Minimum inhibitory concentrations (MIC) and minimum bactericidal/fungicidal (MBC/MFC) concentrations of the *Salvia sclarea* L. extracts and standards (S) on bacterial Gram (+) and Gram (−) strains and a yeast.

Extracts		MM	MU	M80M	M80U	S
Bacterial Strain		MIC/MBC (mg/mL)	MIC/MBC (mg/mL)	MIC/MBC (mg/mL)	MIC/MBC (mg/mL)	MIC/MBC (μg/mL)
Gram (+)	ATCC					Chlor.
*Staphylococcus aureus*	6538	12.5/>100	6.25/>100	12.5/100	25/>100	7.81/15.61
*Streptococcus pneumoniae*	6301	100/>100	100/>100	50/>100	100/100	0.06/0.12
*Streptococcus pyogenes*	19615	>100/>100	>100/>100	100/>100	100/>100	0.25/0.49
*Enterococcus faecalis*	9433	>100/>100	>100/>100	100/>100	100/>100	3.91/7.81
*Bacillus cereus*	11778	25.0/>100	12.5/>100	12.5/>100	50/>100	7.81/15.61
*Listeria monocytogenes*	15313	25.0/>100	25/>100	50/>100	50/>100	0.25/0.49
Gram (−)	ATCC					Str.
*Pseudomonas aeruginosa*	9027	100/>100	50/>100	50/50	100/100	0.60/0.60
*Proteus mirabilis*	12453	>100/>100	>100/>100	100/100	100/>100	0.30/0.30
*Salmonella enteritidis*	13076	100/>100	>100/>100	100/100	100/>100	0.30/0.30
*Escherichia coli*	8739	100/>100	>100/>100	100/>100	100/>100	0.16/0.16
*Enterobacter aerogenes*	13048	100/>100	50/>100	100/>100	100/>100	0.60/0.60
*Klebsiella pneumoniae*	10031	100/>100	100/>100	100/>100	100/>100	0.30/0.30
fungal strain		MIC/MFC (mg/mL)	MIC/MFC (mg/mL)	MIC/MFC (mg/mL)	MIC/MFC (mg/mL)	MIC/MFC (μg/mL)
yeast	ATCC					Nys.
*Candida albicans*	24433	100/>100	100/>100	100/>100	>100/>100	3.91/7.81

Chlor.—chloramphenicol; Str.—streptomycin; Nys.—nystatin.

## Data Availability

Not applicable.
